# Autophagy and Human Neurodegenerative Diseases—A Fly’s Perspective

**DOI:** 10.3390/ijms18071596

**Published:** 2017-07-23

**Authors:** Myungjin Kim, Allison Ho, Jun Hee Lee

**Affiliations:** Department of Molecular and Integrative Physiology, University of Michigan, Ann Arbor, MI 48109, USA; allho@umich.edu

**Keywords:** Drosophila, neurodegeneration, autophagy, Alzheimer, Parkinson, Huntington, ataxia, protein aggregate, mitochondrial dysfunction

## Abstract

Neurodegenerative diseases in humans are frequently associated with prominent accumulation of toxic protein inclusions and defective organelles. Autophagy is a process of bulk lysosomal degradation that eliminates these harmful substances and maintains the subcellular environmental quality. In support of autophagy’s importance in neuronal homeostasis, several genetic mutations that interfere with autophagic processes were found to be associated with familial neurodegenerative disorders. In addition, genetic mutations in autophagy-regulating genes provoked neurodegenerative phenotypes in animal models. The Drosophila model significantly contributed to these recent developments, which led to the theory that autophagy dysregulation is one of the major underlying causes of human neurodegenerative disorders. In the current review, we discuss how studies using Drosophila enhanced our understanding of the relationship between autophagy and neurodegenerative processes.

## 1. Autophagy in Human Neurodegenerative Diseases

### 1.1. Protein Inclusions and Mitochondrial Dysfunction: Hallmarks of Neurodegeneration

Neurodegeneration is a progressive loss of neuronal structure and function, resulting in an irreversible decline in cognitive abilities such as memory and decision-making, as well as bodily coordination and mobility. Of the many different types of neurodegeneration, the most notable and common forms include Alzheimer’s, Parkinson’s and Huntington’s diseases. Although these diseases manifest with distinct clinical features and affect different regions of the brain, they share very similar molecular pathologies at the cellular level, such as the accumulation of misfolded protein aggregates, which are toxic to the cells [1]. More specifically, Alzheimer’s disease is characterized by the extracellular accumulation of β-amyloid protein (Aβ) plaques as well as the intracellular accumulation of tau, a microtubule-associated protein. Parkinson’s disease is similarly characterized by intracellular aggregates of α-synuclein and ubiquitin, known as Lewy bodies. Huntington’s disease also exhibits protein inclusions, consisting of a mutant Huntingtin protein that has a polyglutamine expansion. In addition to the protein aggregates, these diseases also exhibit an accumulation of dysfunctional mitochondria, which produces excessive reactive oxygen species that damage cellular macromolecules such as DNA, lipids and proteins [2]. The accumulation of protein inclusions and damaged mitochondria are often considered histological hallmarks of neurodegeneration.

### 1.2. Genetic Mutations that Lead to Protein Inclusions

How these molecular pathologies develop has remained a mystery for a long time. For some of these diseases, it was clearly shown that a genetic mutation is the primary cause of the disease. Huntington’s disease is caused by a polyglutamine expansion mutation in the gene encoding the Huntingtin protein; the mutant protein with a polyglutamine tract accumulates inside neurons as toxic protein inclusions [3]. Similar polyglutamine expansion mutations are found in other types of neurodegenerative diseases such as several forms of spinocerebellar ataxia [3]. Additionally, for a minor subset of Parkinson’s cases, several genetic mutations were identified as causing the disease by upregulating the α-synuclein-encoding genes, leading to the accumulation and aggregation of the protein [4]. Although these genetic mutations explain protein inclusion pathologies in a small number of patient populations, the predominant portion of Alzheimer’s and Parkinson’s cases do not clearly associate with genetic mutations. This obscures our complete understanding of the etiology of these diseases.

### 1.3. Autophagy—One of the Major Homeostatic Processes to Eliminate Protein Aggregates and Damaged Mitochondria

Autophagy is primarily defined as a bulk degradation system for various cytoplasmic components [5]; however, it can also be selectively directed towards a specific substrate such as aggregated proteins or dysfunctional mitochondria through specific adaptor proteins such as p62/SQSTM1 [6]. Autophagy is initiated by the formation of a phagophore, a double-membrane structure, around the target cargo. The phagophore engulfs the target, forming an autophagosome that sequesters its cargo from the cytosol. The autophagosome then fuses with a lysosome, which contains digestive enzymes to degrade the target cargo. Finally, degradation products are released from channels on the lysosomal membrane. Through this process, autophagy removes toxic protein aggregates, excessive nutrient deposits such as lipid droplets, and dysfunctional organelles such as damaged mitochondria [7,8]. 

Pioneering work in the yeast system has identified a number of genetic components that are critical for the formation of phagophores and autophagosomes [9]. These include the autophagy-regulating genes (*ATGs*). For example, ATG1 (ULK1), which complexes with ATG13 and ATG17 (FIP200), is a protein kinase whose activity is critical for the initiation of autophagy [10]. The Class III phosphatidylinositol 3-kinase (PI3K) complex, composed of Atg6 (BECN1), Vps15, and Vps34, is also important for autophagy initiation [11]. Atg18 (WIPIs) is one of the effectors of the class III PI3K activity in autophagy [12]. Upon ATG1 activation, class III PI3K activation and autophagy initiation, other ATG proteins are required for the formation and elongation of the phagophore [9]. Specifically, ATG9 is required for formation of the phagophore, and ATG5 plays a role in elongation and maturation of an autophagosome. During autophagy, ATG5 is covalently bound to ATG12, a ubiquitin-like protein, by the actions of the E1- and E2-like enzymes ATG7 and ATG10, respectively. ATG5–ATG12 conjugates, complexed with another protein ATG16, are also important for activating the lipidation process for ATG8, mediated by ATG7 and ATG3. Lipidated ATG8 is a marker for active autophagy because it is one of the major constituents of the autophagosomal membrane. All of these ATG proteins are strikingly well conserved throughout the eukaryotic kingdom, and they play a critical autophagy-regulating role in both animals and yeasts [5]. These mechanisms defined by using the yeast system exemplify the capability of genetic analyses in model organisms to approach complicated mechanisms in higher eukaryotes.

### 1.4. Role of Autophagy in Neuronal Homeostasis and Preventing Neurodegeneration

The autophagic removal of protein aggregates and dysfunctional mitochondria could be very critical for non-dividing cells of multicellular organisms, such as neurons and myocytes [7,13,14,15,16,17]. These long-lived cells are expected to accumulate unfolded or misfolded proteins as well as damaged organelles. However, autophagosomes are very scarcely found in neurons either in intact tissue or in primary culture; therefore, autophagy was initially thought to be inactive in neurons. It was later found that autophagosomes are not abundant in neurons because neurons have a highly efficient lysosomal degradation system, which removes autophagosomes rapidly. Accordingly, the inhibition of lysosomal activity in primary cultured neurons led to the accumulation of autophagosomes even in the absence of any autophagy-stimulating signals such as starvation, demonstrating that autophagy is highly active in neuronal cells [18]. Animal models with genetic ablation of core autophagy genes (e.g., *ATG* genes) also revealed a critical function of autophagy in maintaining neuronal health [19,20,21,22,23,24]. Autophagy gene mutations in flies and mice induced prominent deterioration of neuronal health [19,20,21,22,23,24]. Specifically, autophagy-deficient neurons resulted in the excessive accumulation of ubiquitin/p62-positive protein inclusions and damaged mitochondria, cellular hallmarks of neurodegenerative disease [19,20,21,22,23,24]. The presence of protein inclusions and damaged mitochondria in cells leads to neurodegenerative pathologies such as neuronal dysfunction and cell death, as well as subsequent deficits in cognitive function and mobility [19,20,21,22,23,24]. These results suggest that autophagy plays an important role in maintaining neuronal homeostasis and preventing neurodegenerative pathologies [15,16].

### 1.5. Implication of Autophagy in Alzheimer’s Disease

Ultramicroscopic analysis of degenerating brain tissue from Alzheimer’s patients revealed the prominent accumulation of non-degraded autophagic vesicles, indicating that autophagic flux was arrested [25]. Cell or animal models for Alzheimer’s disease also frequently exhibited defective autophagic flux and the accumulation of autolysosomal vesicles. For example, the presenilin 1 (PS1) mutation, which is the most frequent cause of early onset familial Alzheimer’s disease, is known to cause autophagic arrest in both mouse [26,27] and cell culture models [28]. The expression of the amyloid precursor protein (APP) with mutations found in familial Alzheimer’s disease also triggered similar autophagic-lysosomal pathologies [29]. The expression of Aβ, the cleavage product of APP, also induced the accumulation of large autophagic vesicles in the Drosophila brain [30]. This evidence suggests that Alzheimer’s disease involves the defective regulation of autophagy at the autophagosomal degradation step [15].

### 1.6. Implication of Autophagy in Parkinson’s Disease

Parkinson’s disease is another prominent neurodegenerative disease with phenotypes indicative of defective autophagy. Analysis of familial Parkinson’s disease identified several causative genes called *PARK* genes [4]. Most of these genes, including *PARK1 *and* PARK4* (*SNCA*), *PARK2* (*Parkin*), *PARK5* (*UCHL1*), *PARK6* (*PINK1*), *PARK7* (*DJ-1*), *PARK8* (*LRRK2*) and *PARK9* (*ATP13A2*), were found to play some role in the autophagic elimination of ubiquitinated proteins or damaged mitochondria [31,32,33,34]. In addition, diseased tissues from Parkinson’s patients present with protein aggregates and damaged mitochondria [31,32,33,34]. Therefore, autophagy also seems to prevent the progression of Parkinson’s disease pathologies. The Drosophila model has contributed significantly to understanding these *PARK* components, as well as other genetic components associated with neurodegeneration, in the regulation of autophagic processes.

## 2. Drosophila as a Model for Investigating the Autophagy-Neurodegeneration Relationship

### 2.1. Drosophila as a Genetically Tractable Model Organism

Drosophila has been extensively described as a useful genetic model of human diseases [35,36,37]. Historically, the Drosophila model has been proven to be valuable for genetic analyses including genome-scale screening studies. Drosophila has high genomic and physiological homology to mammals, and around 65% of human disease-causing genes are conserved [35,37]. Drosophila also have a rapid life cycle (~2 weeks per generation), high fecundity (one wild-type Drosophila female can lay up to 100 eggs in a day) and relatively low maintenance costs that allow for genome-scale genetic screening [36]. These attributes cannot be matched by any other vertebrate animal model. A number of genetic components in diverse signaling pathways, such as Ras-MAPK, Sonic Hedgehog (Hh), TGF-β (Dpp), Wnt (Wg) and Notch signaling pathways, were identified and characterized originally in the Drosophila model. Most of the signaling molecules identified from genetic screens in Drosophila were shown to be functionally conserved in mammalian organisms in subsequent studies, demonstrating the suitability of Drosophila for studying molecular signaling pathways in animals.

### 2.2. Drosophila as a Model for Investigating Autophagy

The first genetic study of autophagy in Drosophila was published in 2003 [38]. Soon, the Drosophila system proved to be highly efficient for analyzing the autophagy-related signaling network. Using Drosophila genetics, nutrient-regulated target of rapamycin (TOR) signaling [39], insulin/growth factor-regulated PI3K signaling [40,41] and nuclear hormone receptor signaling [41] were demonstrated to be part of the autophagy-regulating network that responds to both environmental and developmental cues. The genetic ablation of *Atg7*, a core autophagy gene, resulted in deterioration of neuronal health and longevity [21], revealing a role for autophagy in physiological homeostasis. Later, we showed that Atg1/ULK1 and Sestrin, another autophagy regulator [10,42,43], are critical for eliminating damaged mitochondria and maintaining functional and structural integrity of cardiac and skeletal muscle [44]. Mutations in other autophagy-related genes have also been shown to provoke neurodegeneration in Drosophila (see Table 1). Importantly, in mammalian systems, these autophagy components are also critical for metabolic (e.g., liver and muscle) and long-lived organs (e.g., neurons) [19,20,45,46]. This suggests that the Drosophila system is highly relevant for investigating the physiological role of autophagy in higher order animals. 

### 2.3. Drosophila as a Model for Investigating Human Neurodegenerative Diseases

**Figure 1:**
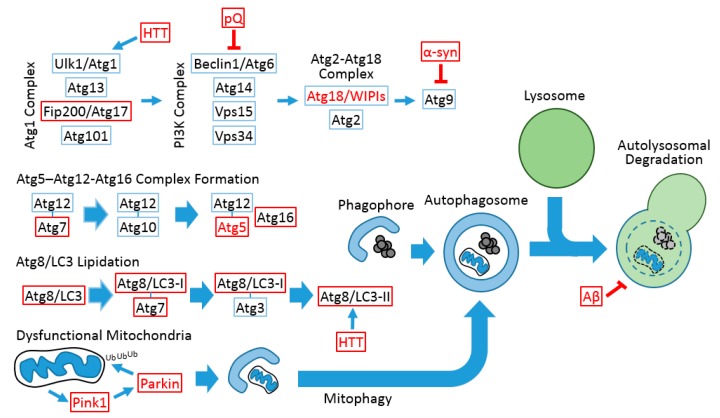
Conserved autophagic processes between Drosophila and mammals. Red letters indicate that the gene and protein are associated with neurodegenerative diseases in humans. Red boxes indicate that the gene mutation and/or transgenic modulation can provoke neurodegeneration or neuronal dysfunction in the Drosophila system. Blue boxes indicate that, although the Drosophila gene is implicated in the autophagic process, its function in neuronal homeostasis has not been assessed. Thin arrows indicate signal flow, and thick arrows indicate the modification of complexes and vesicles. Blunted arrows indicate inhibition signals. The autophagosomal inner membrane and its contents are degraded after lysosomal fusion (dashed circle). pQ, polyglutamine tract; α-syn, α-synuclein; HTT, Huntingtin; Aβ, β-amyloid.

Completion of genome sequencing revealed that approximately 65% of human disease-causing genes are conserved in Drosophila [35,37]. Even in cases where a gene is not found in the Drosophila genome, the function of such a gene can still be investigated in the fly through ectopic expression of the human protein [36]. Genes associated with familial neurodegenerative diseases in humans and proteins associated with disease pathophysiology, such as Aβ, mutated tau (Alzheimer’s disease), α-synuclein, mutated Pink, Parkin and Dj-1 (Parkinson’s disease), mutated Huntingtin and a polyglutamine tract (Huntington’s disease and ataxia) caused neuropathologies in flies that are very similar to what was observed in human patients [52,53,54]. Notably, the mis-expression of some of these components resulted in apparent degeneration of eye photoreceptor cells, which can be non-invasively examined and easily scored for genetic interaction assays [52]. These studies establish Drosophila as a robust model for investigating neurodegenerative diseases, as well as their relationship with autophagy pathways (Figure 1).

### 2.4. Drosophila Models of Alzheimer’s Disease: β-amyloid and Tau Flies and Their Autophagic Phenotypes

β-amyloid (Aβ), a cleavage product of amyloid precursor protein (APP), is the major component of extracellular plaques found in the brain tissue of patients with Alzheimer’s disease. Recent evidence demonstrates that intracellular accumulation of Aβ could precede extracellular accumulation and promote the early pathogenesis of Alzheimer’s disease [55]. Consistent with this intraneuronal role, the expression of Aβ in Drosophila neurons produced prominent degenerative phenotypes [30,56]. These include visible external degeneration of the ommatidia structure in the eyes [30], as well as dramatically reduced lifespan and mobility [56]. Of note, Aβ neurotoxicity is accompanied by a strong arrest of autophagic flux, which was assayed by degradation of exogenously expressed green fluorescent protein (GFP) [30]. Neurons expressing Aβ accumulate large amounts of non-degraded and oftentimes damaged autophagosomes, which are visible through Atg8-GFP reporters as well as through a transmission electron microscope (TEM) [30]. These histological pathologies bear a great resemblance to what is observed in human Alzheimer’s patients [25] and suggest that intracellular Aβ interferes with autophagosome degradation. 

In addition to Aβ, Alzheimer’s disease is also characterized by intracellular accumulation of tau, a microtubule-associated protein, suggesting that tau neurotoxicity could be another important pathogenetic mechanism for the disease [57]. Tau neurotoxicity was also successfully modelled in Drosophila by overexpressing human tau protein [58,59]. Like the Aβ model, tau overexpression produced strong neurodegeneration that provoked ommatidia malformation [59], brain tissue degeneration and reduced lifespan [58]. Several genetic modifier screens were performed using either an overexpression library (enhancer and promoter (EP) lines) [60] or a lethal mutant library (P-lethal lines) [61], which isolated signaling proteins that post-translationally modify tau. Some autophagy components, such as Atg6, were also isolated from the screens [61]. Importantly, upregulation of autophagy significantly reduced tau toxicity [62], suggesting that autophagy also protects neuronal health in this fly model.

For both models, disease-associated pathogenic variants, such as the Arctic mutation (E22G) of Aβ [56] and frontotemporal dementia-associated mutations (V337M and R406W) of tau [58] produced stronger pathogenetic effects in the flies. Furthermore, the expression of both Aβ and tau synergistically accelerated neurodegeneration in Drosophila [63,64]. These findings indicate that the Aβ and tau models in Drosophila are relevant for investigating Alzheimer’s disease and suggest that these neurodegenerative pathologies are closely associated with dysfunctional or dysregulated autophagy.

### 2.5. Drosophila Models of Parkinson’s Disease: α-Synuclein, PARK Genes and How Autophagic Processes are Compromised in These Models

Flies expressing the human α-synuclein (α-syn) gene were one of the first transgenic models in Drosophila to focus on investigating a major human neurodegenerative disease [65]. α-syn is the primary structural component of a Lewy body, an insoluble protein aggregate found in brain tissues of Parkinson’s patients, as well as some other neurodegenerative diseases. Of note, several mutations in α-syn-encoding genes, such as point mutations and gene amplifications, are associated with familial Parkinson’s disease [4]. As in humans, the overexpression of α-syn in Drosophila provoked prominent neurodegeneration associated with Lewy body-like structures, neuronal cell death and mobility impairment [65]. α-syn overexpression also caused neurodegeneration in the Drosophila eye, including degeneration of the rhabdomere structure. Interestingly, α-syn overexpression did not lead to externally visible eye defects, suggesting a much milder phenotype than the models discussed above. Still, α-syn in the Drosophila model was shown to directly interfere with autophagic flux, resulting in a pronounced accumulation of other autophagy substrates, such as mutant Huntingtin protein with a polyglutamine tract (see below) [66].

There are also fly models that reproduce genetic mutations found in familial Parkinson’s disease. These include flies with loss-of-function mutations in *PARK2/Parkin* [67,68,69], *PARK6/Pink1* [70,71], *PARK7/Dj1* [72,73,74] and *PARK8/Lrrk2* [75,76,77], as well as *PARK11/Gigyf* [78] that we recently characterized. Original reports of Drosophila *Parkin* and *Pink1* mutants linked the Parkin and Pink1 proteins with mitochondrial homeostasis [67,68,70,71,79] and later cell biological studies established that these two proteins are essential components for eliminating dysfunctional mitochondria through a selective autophagic process called mitophagy [80,81]. In this process, damaged mitochondria with defective membrane potential recruit Pink1 to their surface. Pink1 then recruits Parkin, which ubiquitinates mitochondrial surface proteins. This ubiquitination then induces the formation of a phagophore around the damaged mitochondria and targets the defective mitochondria for autophagic degradation. This is considered one of the major quality control mechanisms for mitochondria, not only in neurons but also in other tissues such as the liver [82] and heart [83].

Other genes, such as *Dj1*, *Lrrk2* and *Gigyf* were also shown to be critical for autophagic elimination of ubiquitinated proteins or damaged mitochondria, but the mechanism still needs further clarification [31,32,33,34,78]. Although currently uncharacterized, Drosophila also has a single ortholog of *PARK9/Atp13A2*, which in mammals is required for efficient lysosomal degradation of autophagosomes [84,85].

### 2.6. Drosophila Models of Polyglutamine Toxicity: Polyglutamine Flies and Their Genetic Interaction with the Autophagic Pathway

In several inheritable neurodegenerative diseases, known as polyglutamine diseases, genetic mutations include a long polyglutamine (pQ) tract in the coding sequence of a specific gene [3]. For example, Huntington’s disease is caused by a pQ extension in the Huntingtin (*HTT*) gene, while spinocerebellar ataxia (SCA) is caused by a similar pQ extension in various genes including Ataxins (e.g., *ATXN1-3*). The accumulation of proteins with a polyglutamine tract often causes protein aggregation and inclusion, which pathogenetically produces proteotoxicity. Because protein aggregates containing pQ can be eliminated through autophagic machinery, autophagy is important for preventing the development of pQ pathologies. In the Drosophila model, pQ toxicity can be easily modeled by its drastic effects on eye morphology [53]. Genetic modifiers of pQ toxicity in Drosophila include critical autophagy components such as Atg6, Atg12 and Ref(2)P, a p62/SQSTM1 homolog [86,87]. Chemical enhancers of autophagy also alleviated pQ-induced retinal degeneration [88]. Recent cell biological studies showed that pQ also interferes with autophagic flux by downregulating class III PI3K through inhibiting its interaction with Ataxin-3, the gene mutated in SCA3 [89]. Importantly, HTT, whose expression is abrogated by the pQ expansion in Huntington’s disease, was recently found to facilitate selective autophagy by acting as a scaffold between autophagy adaptor p62/SQSTM1 and core autophagy molecules such as Atg1/ULK1 and Atg8/LC3 proteins [90,91]. The role of HTT in autophagy was initially identified from Drosophila genetics, and later confirmed in knockout mice and human cells [90,91]. In other cell biological studies, pQ aggregates were found to interact with ALFY, another scaffolding protein for p62/SQSTM1 [92,93,94]. Mutations in the Drosophila homolog of ALFY, known as *blue cheese* (*bchs*), also provoked progressive neurodegeneration associated with prominent accumulation of insoluble protein inclusions [95,96,97]. Eye-based genetic screening in Drosophila further identified many autophagic components in the *bchs* pathology including: Atg1; Atg2; Atg6; Atg8a, and; Atg18 [95]. All of this information acquired from HTT, pQ and *bchs* flies shows how Drosophila genetics enhances our understanding of the relationship between autophagy and neurodegeneration.

### 2.7. Using Drosophila for Investigating the Pathogenetic Role of Human Autophagy Mutations

Extensive studies established that genetic disruption of autophagy provokes neurodegenerative phenotypes in model animals such as Drosophila and mice, and many mutations that cause neurodegenerative disease in humans compromise the autophagic process. The direct evidence indicating that autophagic defect causes neurodegeneration in humans was also recently provided: a mutation in *ATG5*, a core autophagy-regulating gene, was discovered in two ataxia patients from a consanguineous family [22]. The mutation (E122D) is a subtle amino acid change, but assays in patient cell lines indicated that the mutant ATG5 (ATG5–E122D) has a defect conjugating with ATG12, which is necessary for its autophagy-initiating activity. In addition, the mutant cells exhibit dramatic impairment in autophagic flux, monitored by an LC3-II conversion assay. Furthermore, the mutant cells accumulate higher amounts of p62/SQSTM1, an autophagy substrate. Still, it was unknown whether this subtle mutation indeed produces pathogenesis.

Here, the power of Drosophila genetics was again proven to be extremely useful. From a Drosophila model, the effect of the ATG5–E122D mutation on neuronal autophagy and homeostasis was successfully reproduced [22]. Flies in which Atg5 is substituted with the human mutant form (ATG5–E122D) were found to exhibit severe movement disorder, while flies expressing the wild-type human protein (ATG5-WT) were protected from such degeneration. ATG5–E122D was also inferior to ATG5-WT in preventing protein aggregation and neurodegeneration in the brain tissue of Atg5-deficient mutant flies. The comparable mutation in yeast also produced strong deterioration of autophagic flux [22], suggesting that the role of E122 is critical across different animal species. These results together established the pathogenetic role of the ATG5–E122D mutation, and provided an example of how the impairment of core autophagy machinery directly causes neurodegenerative disease in humans.

It was also previously shown that heterozygotic mutations in Atg18/WDR45/WIPI4 are associated with static encephalopathy of childhood with neurodegeneration in adulthood (SENDA) [98]. The mutation of Atg18 in Drosophila was shown to impair autophagy through Atg9 mis-regulation [49], although its role in neuronal homeostasis has yet to be examined. Considering that Atg18 family proteins have important roles in mediating the effect of the class III PI3K complex [12], this is another example that highlights the role of autophagy in human neurodegeneration.

## 3. Conclusions and Future Directions

The Drosophila model has been successful in modelling human neurodegenerative disease and investigating physiological roles and regulation of autophagy. These findings, combined with studies in other model systems, such as cultured cells and mice, established the theory that autophagic homeostasis is critical for preventing neurodegenerative phenotypes. Still, there are many different areas where the Drosophila model can be used for future studies in investigating the relationship between autophagy and neurodegeneration.

First, it is not currently known why genetic mutations in autophagy components preferentially deteriorate cerebellar motor function compared to other cognitive functions of the brain. It is most likely that cerebellar neurons are somehow hypersensitive to neurotoxic insults caused by autophagy dysregulation. The preferential motor deficit phenotypes were observed in autophagy-defective human [22], mouse [19,20,24] and Drosophila [21,22,23]. Therefore, the Drosophila model could again prove to be useful for discovering the mechanistic basis of this pathophysiology. How different molecular pathologies in autophagy affect different areas of the brain, causing distinct clinical presentations of neurodegeneration, should also be clarified. As reviewed above, different neurotoxic proteins compromise autophagy through distinct mechanisms in both Drosophila and mammalian cells, which may be the reason why they affect different regions of the brain.

In addition, future studies aimed at isolating additional autophagy-regulating components, through genome-level functional screening in Drosophila, may lead to the construction of a systems-level view of the autophagy network that fine tunes neuronal homeostasis. The construction of this network may enable us to approach the molecular etiology of sporadic Alzheimer’s and Parkinson’s disease, which cover a predominant portion of neurodegeneration cases in humans, yet are not associated with clear genetic mutations. It is possible that age-dependent deterioration of neuronal homeostasis, associated with some subtle genetic predisposition and environmental influence, contribute to the dysregulation of autophagic machinery. The Drosophila model was also recently found to be useful for investigating the effect of aging [99], external insults [100], and subtle genetic alteration [22] on autophagic pathway and neurodegeneration. Therefore, it will provide an efficient platform for investigating the interaction between aging, environments and genetics in the context of neurodegeneration.

These lines of study are expected to enhance our understanding of how autophagic defects contribute to neurodegenerative pathologies. These advancements may ultimately lead to the discovery of novel therapeutics that can restore autophagic flux in diseased neurons and their associated cells. For instance, several chemicals such as rapamycin and calcium channel blockers were shown to enhance autophagy and attenuate neurodegeneration in the Drosophila model [47,62,86,88,101], and similar beneficial effects of these chemicals were observed in different mouse models [101,102,103]. Therefore, further exploration of this autophagy-neurodegeneration network in flies might uncover important pathogenetic mechanisms that can be used for developing clinically-relevant therapeutic strategies to treat human neurodegenerative disease.

## Figures and Tables

**Table 1:** Autophagy gene mutations in Drosophila that provokes neurodegenerative phenotypes.

Gene	Neurodegenerative Phenotype(s) Exhibited by Null Mutants or Hypomorphs
*Atg5*	Defective locomoter activity, p62/Ref(2)P accumulation in brain, neuronal cell death [22]
*Atg7*	Defective locomotor activity, ubiquitin accumulation in brain, neuronal inclusion body formation, neuronal cell death, brain vacuolization [21]
	Retinal degeneration [47]
*Atg8a*	Ubiquitin accumulation in brain [48]
	p62/Ref(2)P inclusion formation in neurons [49]
*Atg16*	Neuronal inclusion body formation, p62/Ref(2)P accumulation in brain [50]
*Atg17/Fip200*	Defective locomotor activity, defective wing posture, ubiquitin accumulation in brain, neuronal inclusion body formation, mitochondrial dysfunction, brain vacuolization [23,51]

## Abbreviations

α-syn	α-synuclein
Aβ	β-amyloid
APP	amyloid precursor protein
ATG	autophagy-related
FIP200	FAK family kinase-interacting protein of 200 kDa
MAPK	mitogen-activated protein kinases
PARK	Parkinson Disease
PI3K	phosphatidylinositol 3-kinase
TOR	target of rapamycin
ULK1	Unc51-like protein kinase 1
